# Re-evaluation of the relationship between paranormal belief and perceived stress using statistical modelling

**DOI:** 10.1371/journal.pone.0312511

**Published:** 2024-11-13

**Authors:** Kenneth G. Drinkwater, Andrew Denovan, Neil Dagnall

**Affiliations:** 1 Department of Psychology, Faculty of Health and Education, Manchester Metropolitan University, Manchester, United Kingdom; 2 School of Psychology, Faculty of Health, Liverpool John Moores University, Liverpool, United Kingdom; The John Paul II Catholic University of Lublin, POLAND

## Abstract

Recent research indicates that paranormal belief, in the absence of allied cognitive-perceptual and psychopathology-related factors, is not associated with negative wellbeing outcomes. However, investigators have historically reported relationships between specific facets of belief (e.g., superstition) and stress vulnerability. These typically derive from the Revised Paranormal Belief Scale (RPBS), which has questionable psychometric integrity. The main issue being that several RPBS items perform poorly. Noting this, the present paper re-examined the relationship between paranormal belief and stress using the Rasch purified version of the RPBS. This comprises two dimensions, called Traditional Paranormal Belief (TPB) and New Age Philosophy (NAP). These are operationalised in terms of function. Specifically, whether belief provides a sense of control at the social (TPB) or individual level (NAP). Accordingly, this study examined whether TPB and NAP were differentially predictive of levels of perceived stress. In this context, stress served as an indicator of well-being. A sample of 3084 participants (*M*age = 50.31, *SD* = 15.20, range 18–91) completed the RPBS alongside the 10-item Perceived Stress Scale (PSS-10). Confirmatory factor analysis and structural equation modelling revealed that TPB was significantly predictive of higher Distress, and lower Coping. NAP was neither predictive of Distress nor Coping. These findings support the notion that TPB is attendant with external control, particularly the notion that unknown supernatural forces/powers influence existence.

## Introduction

Research into paranormal belief is important because supernatural credence persists within contemporary society [1] and potentially influences everyday attitudes and behaviour [2]. For instance, investigators report that paranormal belief is associated with lower levels of trust in science [3] and higher anti-science attitudes [4]. This, in part, explains why believers are more likely to endorse epistemically suspect beliefs. These are notions, not based upon reasoned or reliable evidence, which conflict with prevailing conceptions of the world [5]. Specific examples allied to belief in the paranormal are endorsement of alternative medicine [6], anti-vaccination [3], and conspiracies [7].

Despite such findings, lack of conceptual and methodological coherence limit appreciation of the individual and social impact of paranormal belief. A particular issue being the use of multiple definitions [8]. Noting this, the present paper, adopted the classification of a paranormal belief “as a proposition that has not been empirically attested to the satisfaction of the scientific establishment but is generated within the non-scientific community and extensively endorsed by people who might normally be expected by their society to be capable of rational thought and reality testing” [8, p16-17].

This elucidation is theoretically important because it combines absence of scientific validation with flawed cognitive processing, without the presumption of psychopathology or dysfunction. Moreover, this operationalisation is consistent with the observation that supernatural credence is common within general samples, and acknowledges the fact that believers are not typically maladjusted nor prone to cognitive deficits. This interpretation also aligns with evidence, which suggests that paranormal belief is a form of non-clinical delusion arising from an overreliance on emotional content and the failure to rigorously evaluate the validity of information [9–11].

The concurrent existence and use of a range of instruments and indices to measure paranormal belief further demonstrate lack of theoretical coherence [12]. The two most prominent measures being the Australian Sheep Goat Scale (ASGS, [13]) and the Revised Paranormal Scale (RPBS; [14]) [15, 16]. Traditionally, parapsychologists utilise the ASGS as it centres on traditional core elements of belief (i.e., extrasensory perception, psychokinesis, and life after death) and concomitantly evaluates experience and ability. Whereas social scientists typically employ the RPBS because it samples a broad range of domain content (i.e., supernatural phenomena). A further advantage of the RPBS is that the instrument, via its subscales, appraises different facets of belief (i.e., Traditional Religious Belief, Psi, Witchcraft, Superstition, Spiritualism, Extraordinary Life Forms, and Precognition). Despite dissimilarities, scores on ASGS and RPBS correlate highly indicating that both instruments assess the same underlying construct.

Nevertheless, despite emerging as the predominate research tool, debates remain about the factorial structure of the RPBS. The most used scoring system is unidimensional (i.e., overall score), although researchers occasionally use the original seven subscales, and an alternative two factor model [15]. Lange et al. [17] produced the two-factor RPBS using a top-down purification approach, which combined factor analysis and Rasch scaling [18]. Rasch scaling is a special case of item response theory that addresses frequently cited criticisms of traditional classical test theory (i.e., the true score model). Particularly, the assumption that raw scores, plus or minus random measurement error, accurately reflect test-taker ability. This notion is problematic because error can systematically arise from variations in item difficulty. Hence, test-takers regardless of ability are more likely to endorse or reject certain items. These are items that by virtue of low/high endorsement fail to correctly distinguish between ability levels and falsely decrease/increase overall totals. These items fail to meaningfully contribute to measurement. Noting this, Rasch modelling computes item difficulty. A related issue is differential item functioning (DIF), which occurs when individuals with the same latent ability but from distinct groups (e.g., age and gender) have an unequal probability of giving a response. In this context, group membership rather than ability affects item endorsement.

Although preliminary statistical evaluation reproduced the seven-factor structure advocated by Tobacyk [14], it also identified issues with item functioning. Specifically, multiple items were non-additive and/or displayed DIF related to age, gender, or both. Removal of poorly performing items yielded two correlated unidimensional clusters, which the researchers named Traditional Paranormal Beliefs (TPB, 5-items measuring customary supernatural concepts: traditional religious belief and witchcraft) and New Age Philosophy (NAP, 11-items assessing paranormal abilities: psi, spiritualism, and precognition). In addition to being free from bias, based on anthropological insights [19, 20], these factors represent distinct functions. Specifically, whether the cluster imparts a sense of control at the social (TPB) [21] or individual level (NAP) [22]. Accordingly, culture reinforces TPB, and personal experience strengthens NAP [23].

Commensurate with this interpretation, TPB and NAP characterise distinct worldviews. TPB is associated with fundamental fear of the paranormal and a mechanistic perception of life (i.e., anxiety that uncontrollable forces govern/influence existence), and NAP correlates with supernatural beliefs and experiences that reflect individualism, sense of self-control, and sensation-seeking (i.e., drug use). Consistent with this interpretation, Houran et al. [23] found differing relationships between TPB and NAP and clinical measures. Explicitly, TPB correlated only with the cognitive-perceptual dimension of schizotypy [24] (positive symptoms of psychosis: ideas of reference, magical thinking, and unusual perceptual experiences), whereas NAP correlated with both the cognitive-perceptual and disorganised (i.e., thought disorder and odd behaviour and speech) factors.

### Present study

The lack of coherence caused by the application of different RPBS models, combined with the use of multiple scales to assess belief, has limited the generality of findings across studies. In the area of mental health, the use of a range of alternative measures to assess psychological adjustment and well-being exacerbates this problem. This conceptual concatenation may explain why studies examining relationships between paranormal belief and well-being have produced inconsistent findings.

Consequently, the notion that supernatural credence is associated with maladaptive psychological outcomes is largely predicated on early research based on superstitious belief [25, 26] and not supported by recent work, which indicates that paranormal belief is benign in the absence of high scores on cognitive-perceptual (i.e., transliminality) and psychopathology-related factors (i.e., schizotypy, and manic-depressive experience) [27, 28]. For instance, Dagnall et al. [29] identified transliminality as a connecting variable between paranormal belief, positive schizotypy, and psychopathology. This linking effect is attributable to the fact that higher levels of transliminality reflect hypersensitivity to psychological material (unconscious, and/or external) [30]. Thus, high transliminality increases involuntary susceptibility to ideational and affective phenomena, heightening vulnerability to psychopathology. This relationship is ascribable to key features of transliminality (e.g., reduced latent inhibition, [31]; and lower cognitive flexibility, [32]), which present in psychosis.

While these outcomes indicate that supernatural credence is not detrimental to psychological adjustment and well-being, it remains uncertain whether variations exist because of functional disparities in TPB and NAP. Noting this, the present paper explored whether TPB and NAP were differently associated with perceived stress. Stress in the present study, referred to the degree to which individuals view life as unpredictable, uncontrollable, and overloading [33]. Explicitly, whether participants felt able to cope (respond positively) with and/or found stress distressing (react negatively). Whilst coping and distress are related, there is evidence that these two constructs represent discrete responses [34, 35]. The observation that stress is a frequently used indicator of well-being, which correlates with facets of paranormal belief (e.g., superstition, [36, 37], spiritualism [37], precognition [37], and magical thinking [38]), informed selection of the construct.

The fact that facets of supernatural credence correlate with stress concurs with the psychodynamic hypothesis, which postulates that belief provide a sense of illusory control over external events, and in doing so represent a form of coping [8, 39, 40]. Congruent with this notion, studies report increased levels of superstition [41] and magical thinking [38] during periods of acute societal pressure. However, in such circumstances belief is non-adaptive because despite providing situational reassurance, it encourages avoidant coping, which is associated with lower levels of psychological functioning [42].

Notwithstanding these findings, it is evident that not all facets of belief correlate with stress. Determining which factors do, has previously proven difficult because researchers have either employed a unidimensional RPBS solution, or based analysis on the questionable seven factor solution. This approach is problematic because it derives from poorly performing items and focuses on the content, rather than purpose of belief. Acknowledging this and the sense of control-based operationalisation used by the two-factor model [17, 23], the present paper assessed whether differences in belief function influenced perception of stress.

The researchers measured this using the Perceived Stress Scale (PSS) [43]. The instrument contains general rather than event-specific items, which assess stress in terms of current circumstances and background extraneous influences. Particularly, the degree to which respondents view life as unpredictable, uncontrollable, and overloading [33]. This study employed the 10-item version (PSS-10) because the instrument comprises two factors, distress, and coping [35, 44]. Distress refers to negative affective responses, and coping denotes the capacity to manage/handle stress [44, 45]. Moreover, Rasch scale analysis has revealed no systematic DIF in the PSS-10 (i.e., ethnicity, gender, education, and sample population) [46].

Although this study was exploratory, the researchers hypothesized that since TPB reflects the anxiety that external uncontrollable forces govern and the desire to instil control, it would be a stronger predictor of coping and distress than NAP, which centres on control at an individual level.

## Materials and methods

### Participants

The sample comprised 3084 participants (*M*age = 50.31, *SD* = 15.20, range 18 to 91). There were 1434 males (*M*age = 54.97, *SD* = 14.58, range 18–88), 1638 females (*M*age = 46.27, *SD* = 14.56, range 18–91), 10 non-binary respondents (*M*age = 44.50, *SD* = 16.61, range 25–71), and two preferred not to disclose gender (*M*age = 49.00, *SD* = 1.41, range 48–50). The researchers recruited participants through Bilendi, who are an acknowledged supplier of representative samples [47]. Bilendi provide respondents from a pool of individuals consenting to participate in survey-based research. Appraisal of these data indicated that it is comparable with traditional methods (i.e., researcher-recruited) [48]. The only criteria for inclusion were that participants must be at least 18 years of age and located in the UK. The researchers also requested an equal distribution of preferred gender.

### Materials

The study used established, psychometrically attested, self-report measures.

#### The Revised Paranormal Belief Scale (RPBS)

The RPBS is a 26-item, multidimensional instrument that assesses endorsement of facets of supernatural credence (i.e., Superstition, Psi, Precognition, Traditional Religious Belief, Spiritualism, Witchcraft, and Extraordinary Life Forms) [14]. Items appear as statements (e.g., “The horoscope accurately tells a person’s future”) and participants record their responses on a 7-point Likert scale (1 = strongly disagree to 7 = strongly agree). The RPBS possesses satisfactory validity and reliability at both subscale and global levels [15].

Despite this, Rasch analysis correcting for poor item functioning, identified a purified two-factor model comprising Traditional Paranormal Beliefs (TPB) and New Age Philosophy (NAP) [17]. TPB measures endorsement of core, supernatural concepts such as the devil, hell, and witchcraft, and NAP assesses belief in contemporaneous paranormal abilities (e.g., capacity to mentally influence the physical world, psychokinesis; and predict future events, precognition) and states (e.g., alternative forms of consciousness, astral projection, and spirits) [17]. This hierarchical distinction aligns conceptually with the development of supernatural beliefs (historical/established vs. eclectic concepts, practices, and ways of life).

Psychometrically, TPB and NAP have demonstrated good internal reliability [49–51]. To calculate the two Rasch dimensions it is necessary to convert scoring to 0 to 6 [8], discard non-productive and differentially functioning items (i.e., TPB comprises 11-items and NAP 5-items; see S1 Table for item content), and transform raw to Rasch scaled scores [17].

#### Perceived Stress Scale

The Perceived Stress Scale (PSS-10) measures personal assessments of stress during the past month [34]. Items appear within the scale as statements (e.g., “How often have you felt confident about your ability to handle your personal problems?”) and participants record their responses on a 5-point Likert scale (0 = never to 4 = very often). While researchers often total items to produce an overall score, the present study used the two-factor solution, which comprises Distress and Coping [35]. Distress references negative affective reactions to stress, and Coping indexes the ability to deal with stress [44, 45]. The PSS-10 possesses adequate reliability and validity [35].

### Procedure

Participants retrieved the information sheet via a web link. Only participants who were eligible and agreed to take part progressed to study measures. This involved providing informed consent by clicking a box verifying that they understood the nature of the study. The survey included a brief, demographic section (i.e., asking about age and preferred gender) and the scales (i.e., RPBS and PSS-10). To prevent order effects, scale sequence varied across participants. To counter common method variance, survey instructions emphasised that each section/measure assessed a discrete construct. This approach created psychological distance between measures and lessened the likelihood of response contamination [52]. Finally, to reduce social desirability, additional instructions informed participants that there were no correct answers and directed them to take their time. After completing the survey participants received the study debrief.

The researchers produced this paper as part of a larger, longitudinal, multiphase research project focusing on psychopathology, cognitive-perceptual characteristics, paranormal belief, and wellbeing. This study was unique in terms of its analytical strategy, which assessed relationships using structural equation modelling (SEM) [53]. SEM is a powerful technique because it incorporates a thorough assessment of measurement error by focusing on the item level (as opposed to the variable level) [50]. Additionally, the paper focused on perceived stress as a well-being outcome. In this context, this paper tested different hypotheses to allied scholarly work.

### Ethics statement

Ethical approval was issued by the Manchester Metropolitan University Faculty of Health, Psychology and Social Care Ethics Committee (December 2020; Project ID, 2590). The committee granted permission to undertake the project. Participant recruitment commenced on 29/12/2020 and ran until 29/12/2021.

## Results

### Preliminary analysis

Data screening occurred prior to computing descriptive statistics, involving examination of normality alongside outliers. Then, confirmatory factor analysis assessed adequacy of the measurement model alongside composite reliability. Finally, SEM evaluated relationships between RPBS factors (TPB and NAP) and PSS-10 factors (Distress and Coping).

A range of indices determined model adequacy: chi-square statistic (χ^2^), Comparative Fit Index (CFI), Tucker-Lewis Index (TLI), Standardized Root-Mean-Square Residual (SRMR), and Root-Mean-Square Error of Approximation (RMSEA). CFI and TLI values of .90 and greater are satisfactory [54]. SRMR and RMSEA values of .05 specify good fit, values between .06-.08 indicate satisfactory fit, and between .08 to .10 designate marginal errors of approximation [55]. RMSEA reporting included the 90% confidence interval (CI). For comparison among tested models, analysis used Akaike’s Information Criterion (AIC; [56]), with lower values indicative of better fit.

### Main analysis

Skewness and kurtosis values were in the recommended range −2 to +2 [57] (Table 1). Following the guidelines of Gignac and Szodorai [58], correlation analyses revealed PSS-10 Total and Distress correlated moderately with TPB and NAP. Comparable results occurred in relation to RPBS (Raw) (comprising raw total scores, summed using all 26 items) and RPBS (TPB + NAP) (containing the summed items from TPB and NAP). Coping demonstrated a small correlation with TPB, RPBS (Raw), RPBS (TPB + NAP), and a non-significant association with NAP. Accordingly, model testing assessed only significant preliminary relationships in a predictive capacity (i.e., did not include NAP as a predictor of Coping).

**Table 1:** Descriptive statistics and correlations among study variables.

Variable	*M*	*SD*	Skew.	Kurt.	1	2	3	4	5	6	7
1. PSS-10	16.65	7.85	.12	-.20		.89**	-.68**	.26**	.24**	.23**	.21**
2. Distress	9.27	6.01	.22	-.70			-.29**	.30**	.27**	.26**	.25**
3. Coping	7.38	3.59	.34	.01				-.07**	-.06**	-.09**	-.03
4. RPBS (Raw)	57.68	33.71	.06	-.81					.93**	.88**	.87**
5. RPBS (TPB + NAP)	43.89	11.04	-.66	.30						.94**	.93**
6. TPB	22.71	6.01	-1.18	.96							.76**
7. NAP	21.18	5.76	-.27	.66							

*Note*. PSS-10 = 10-item Perceived Stress Scale, RPBS = Revised Paranormal Belief Scale, TPB = Traditional Paranormal Beliefs, NAP = New Age Philosophy

**p < .001

### Confirmatory factor analysis

The two-factor RPBS model reported unacceptable model fit on all indices but SRMR, χ^2^ (102) = 5733.51, *p* < .001, CFI = .86, TLI = .85, SRMR = .05, RMSEA = .13 (CI of .13 to .14). This aligned with preceding research reporting unacceptable to marginal fit [49, 59]. Permitting correlations among error terms between items 8 and 22, 5 and 12, and 7 and 14 resulted in satisfactory fit, χ^2^ (99) = 3107.03, *p* < .001, CFI = .93, TLI = .91, SRMR = .03, RMSEA = .09 (CI of .08 to .09). Comparison of AIC further supported the superiority of this model (i.e., 3213.03 vs. 5833.51). Statisticians caution against correlation of error terms because it can facilitate chance capitalisation [59]. The exception being when a convincing rationale exists [57]. Assessment of these items revealed that they belonged to shared subscales originating in the seven-factor RPBS model [14]. Moreover, Dagnall et al. [59] found content related items required within-error correlation. Therefore, correlating these error terms was consistent with previous research and facilitated interpretation.

Additionally, the suitability of this model was evident from consulting parameter estimates. All items loaded significantly and positively, and apart from item 23 (loading of .23), loaded above .60, matching the strict conditions of Hair et al. [60]. The theoretically informed two-factor solution for the PSS-10 reported satisfactory model fit across indices, χ^2^ (34) = 563.79, *p* < .001, CFI = .97, TLI = .96, SRMR = .03, RMSEA = .07 (CI of .06 to .07). Furthermore, items loaded positively, significantly, and > .60.

### Composite reliability

Within a latent modelling context, common assessments of reliability (e.g., Cronbach’s *α*) typically under or overestimate reliability [61]. Composite reliability, however, provides a more precise reliability estimate with scores > .60 satisfactory [62]. The TPB (*ρc* = .84) and NAP factors (*ρc* = .93) exhibited satisfactory composite reliability. Similarly, Distress (*ρc* = .92) and Coping (*ρc* = .84) were satisfactory.

### Model evaluation

**Fig 1:**
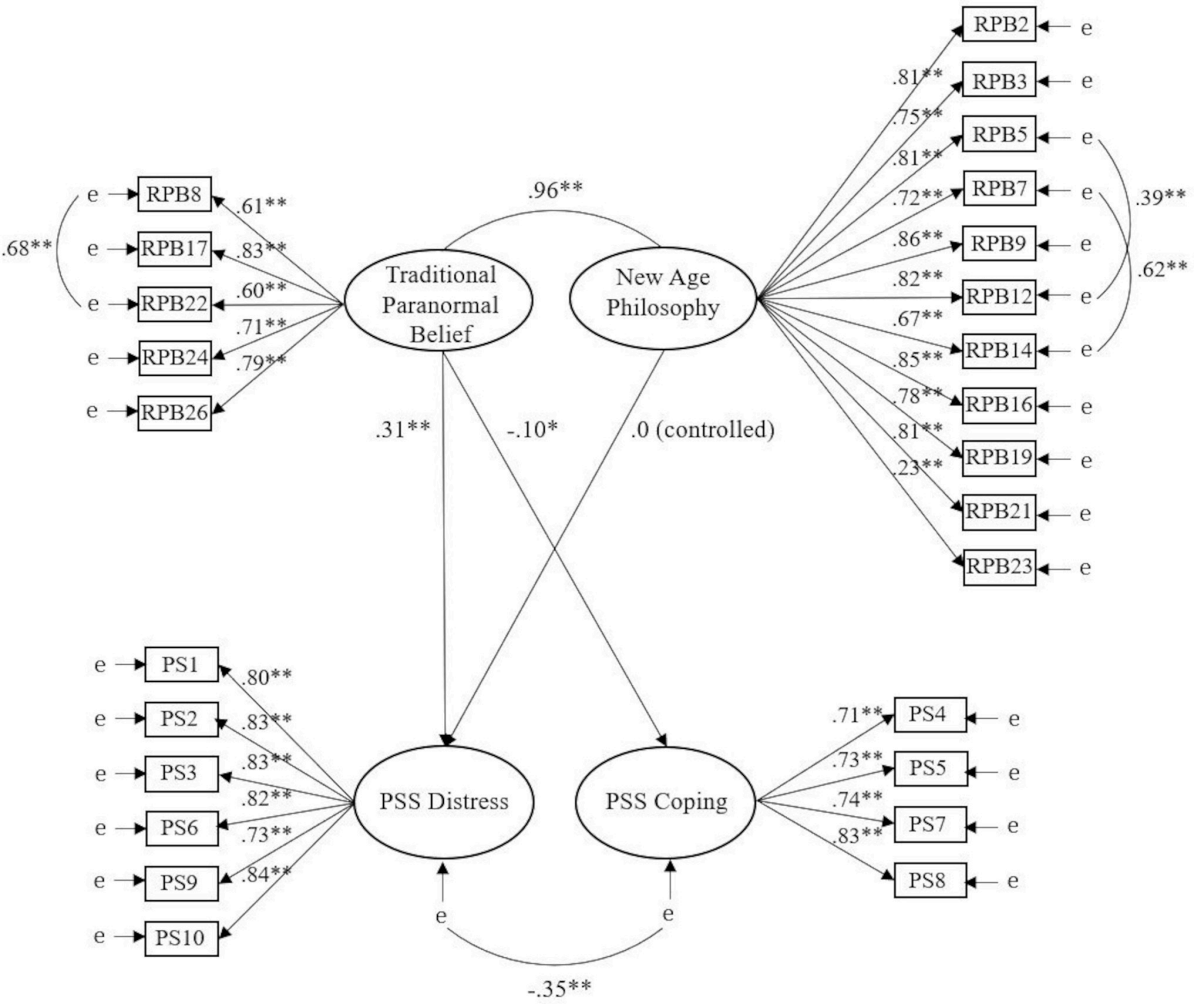
Relationships of RPBS factors with Distress and Coping. *Note*. Ellipses represent latent variables; rectangles represent observed variables; ‘e’ indicates error of measurement; * indicates *p* < .05, ** indicates *p* < .001. PSS = Perceived Stress Scale, RPB = Revised Paranormal Belief, PS = Perceived Stress.

Assessment of data fit for the hypothesised model was satisfactory across indices, χ^2^ (291, *N* = 3090) = 4448.54, *p* < .001, CFI = .93, TLI = .92, SRMR = .03, RMSEA = .06 (CI of .06 to .07). Scrutiny of parameter estimates revealed that TPB was a significant positive predictor of Distress (*β* = .39, *p* = .009) and a significant negative predictor of Coping (*β* = -.10, *p* = .003). NAP did not significantly predict Distress (*β* = .08, *p* = .585). Reanalysis controlling for the non-significant path between NAP and PSS-10 Distress found similar model fit, χ^2^ (292, *N* = 3090) = 4447.84, *p* < .001, CFI = .93, TLI = .92, SRMR = .03, RMSEA = .06 (CI of .06 to .07). However, lower AIC existed (4618.84 vs. 4620.54), specifying superior fit for the refined model. Parameter estimates (Fig 1) indicated that TPB remained a significant positive predictor of Distress (*β* = .31, *p* < .001) and a significant negative predictor of Coping (*β* = -.10, *p* = .003). The model explained 4% of variance in Coping and 9.6% of variance in Distress.

## Discussion

Replicating previous investigations (see [37, 63], scores on the RPBS and Perceived Stress Scale correlated positively. Additionally, consistent with the notion that RPBS dimensions serve distinct psychological functions (i.e., social vs. individual control) [17, 23], TPB significantly predicted greater Distress and lower Coping, whereas NAP predicted neither. From a psychological perspective, these findings corresponded with the supposition that TPB, because it reflects concerns and anxieties about lack of control over external forces, is associated with higher levels of perceived stress. In this context, TPB outcomes aligned with earlier research, which views endorsement of customary supernatural concepts such as religion and magical thinking as ineffective attempts to make sense of the external world [8, 21, 64].

Outcomes require cautious interpretation because correlation-based analysis does not establish cause and effect relationships. Thus, it is also possible that higher levels of stress increase TPB. Since the present study found a link between TPB and Perceived Stress, subsequent research using experimental manipulations and multiple time points should establish cause and effect. Nonetheless, the stable nature of paranormal belief and previous theoretical rationalizations (see Irwin, [8, 64]) suggest that TPB is reflective of reduced coping efficacy. Furthermore, TPB and Perceived Stress reflect reduced, subjective sense of control over external factors. Correspondingly, studies have demonstrated that low sense of control is significantly associated with greater paranormal belief. This is true even when analysis constrains factors conceptually related to sense of control (i.e., demographic characteristics, and paranormal experiences and exposure) [65].

In the case of TPB, Houran et al. [23] contend that the construct reflects fear of the paranormal and a mechanistic perception of life, which views existence as governed by uncontrollable forces. Such attributions are unlikely to reduce stress as they reflect apprehensions about the world and dispose believers to ontological confusions, where core characteristics of mental, physical, and biological entities/processes become concatenated [66, 67].

Moreover, since culture shapes and reinforces TPB, the degree to which TPB provides a sense of control over exterior factors is likely to vary in accordance with the degree to which belief is internalised. Thus, TPB may serve as a buffer against stress if the individual possesses strong conviction. For instance, religious credence arises for myriad reasons (inculcation, rituals, etc.), which are not compatible with the notion that spiritual forces resolve real world problems. Accordingly, while TPB may increase in times of stress, it does not necessary instil a sense of control, nor does it address sources of concern. In circumstances where supernatural credence affords false comfort/reassurance and/or prevents individuals from tackling stressors, researchers have viewed beliefs as a form of non-adaptive, avoidant coping.

This interpretation is congruent with the definition of TPB as a response to uncontrollable forces. It acknowledges that whilst TPB is needs-serving (i.e., provides meaning), the explanations offered do not counter stress because they are deterministic (e.g., the will of powerful sacred being and/or magical/mystical forces) and encourage passivity. Although this elucidation concurs with the observation that TPB was associated with greater Distress and lower Coping, investigators should conduct further research to establish its generality to specific real-life contexts.

A related extension is to examine whether TPB is more strongly related to avoidance (vs. approach) coping than NAP. Avoidance coping strategies are maladaptive as they circumvent dealing with stressors and produce disengagement /lack of volition [68]. In contrast, approach coping strategies are adaptive because they are purposeful, problem-focused, and involve active support seeking (such as drawing on social sources for instrumental and emotional assistance) [68]. Accordingly, assessing whether TPB predicts coping style would further prior research, which has found that general paranormal belief [69] and endorsement of conspiracy theories [70] are positively related to avoidance coping.

Another factor to consider is whether there are differences in TPB as a function of high (vs. low) scoring believers. Scores for TPB and NAP in the present study were low and reflected superficial, rather than deep faith. Although, in comparison to many paranormal-related studies this paper recruited a large sample comprising a fairly equal gender split alongside a good range of ages. Even so, subsequent investigations should examine whether TPB and NAP in high scoring samples perform differently. Certainly, at a conceptual level, conviction merits evaluation alongside belief endorsement.

The use of the two-factor model makes it difficult to draw comparisons with previous studies examining relationships between paranormal belief and stress. Firstly, because studies have typically used the original seven factor model and/or employed the RPBS as a global measure [37]. Secondly, investigations have studied only specific facets of belief (e.g., superstition and magical thinking) [36, 39]. Noting this, ensuing research should compare the two-factor solution with these previous known correlates. This is especially necessary as the two-factor model excludes RPBS items assessing superstition. Hence, evaluating superstition alongside TPB and NAP would prove a worthwhile follow-up study.

A further limitation with the application of the two-factor model is that although studies have reported differences between TPB and NAP [49, 71], other investigations have failed to consistently replicate these findings [59]. Given that the two factors correlate highly, this suggests that they may not be as functional discrete as originally conceptualised. Hence, researchers need to undertake more work to determine the convergent and divergent validity of TPB and NAP. Additionally, preceding investigation should examine whether the two factors are differentially predictive of other related measures such as the Psychological Wellbeing Scale [72, 73], which measures six aspects of well-being and happiness (i.e., autonomy, environmental mastery, personal growth, positive relations with others, purpose in life, and self-acceptance).

Although the outcomes concurred with preceding studies, the use of a cross-sectional design afforded only limited insights. This is important to note, when considering the paranormal belief-stress relationship since the constructs interact in complex ways. Although beliefs once formed are trait-like and remain stable over time [69], there is evidence to suggest that they can vary because of acute stress [37, 39]. Moreover, stress changes rapidly in accordance with alterations in internal and external circumstances.

These factors indicate that single snapshots in time will be unable to fully capture the dynamic nature of the paranormal belief-stress relationship. Recognising this limitation, subsequent research should measure levels of paranormal belief and stress at multiple time points across over an extended period. Outcomes will establish the temporal stability of relationships and inform causal inferences [74]. This is also necessary as the present study only examined perceived stress over one month. Typically, for stress to be harmful it needs to be prolonged.

Moreover, a potential issue with the use of self-report measures is the risk of low-quality responses due to issues such as participant inattention. It would be useful for future research to control for this using remedial techniques such as attention checks (e.g., as practiced with popular psychometric scales including the HEXACO-PI-R [75]). It is though important to note that thorough data screening in the present study reduced the likelihood of low-quality responses affecting outcomes.

A limitation of the RPBS is that it employs a broad definition of the paranormal, which classifies a range of non-scientific beliefs as supernatural. The problem with this delineation is that it subsumes beliefs typically associated with religious traditions and beliefs not typically associated with (religious traditions). These beliefs also reflect a Western, Abrahamic, predominately Christian perspective. Within the two-factor model, religious supernatural beliefs appear within TPB and non-religious supernatural beliefs within NAP. This categorisation overlooks the intuitive, layperson distinction between religious (soul, the devil, God, and heaven and hell) and paranormal (psi, witchcraft, superstition, etc.) beliefs.

Accordingly, Baker et al. [76] introduced the concept of bounded affinity. This acknowledges that despite inherent similarities (i.e., physiological, psychological, and ontological), organised religious groups differentiate between a narrow subset of acceptable (true) and unacceptable (false) experiences and explanatory frames. This proposes an alternative definition of the paranormal as those beliefs and experiences rejected by science and organized religions. This operationalisation of the paranormal explains why, as a function of cultural and empirical contexts, religions hold negative, positive, or non-linear relationships to paranormal phenomena. Noting this, subsequent studies should assess whether religious truth versus supernatural beliefs interact differently with stress and wellbeing outcomes.

The finding that only TPB was associated with higher levels of perceived stress, is relevant to clinical contexts since it suggests that targeting these beliefs reduces or helps individuals experiencing elevated levels of distress and lower coping. TPB, unlike NAP, which is more strongly associated with psychopathology or adverse personality structure (i.e., dissociative, and schizotypal tendencies) is socially, culturally oriented. Accordingly, TPB allied issues link to external rather than personal factors. Noting this, Houran et al. [23] referred to TPB as surrogate religious beliefs acquired through social learning. Hence, TPB represents a strongly internalised representation of the external world, which focuses on irrepressible supernatural powers and forces. This delineation potentially explains the connection between TPB and stress susceptibility. Noting differences between TPB and NAP, Houran et al. [23] proposed that the two constructs reflect the distinction between paranormal and religious belief.

Commensurate with this demarcation, TPB and NAP influence the formation and maintenance of delusions in particular ways. Explicitly, predominant beliefs influence attributional processes and shape credence-related cognitions and perceptions. Hence, TPB reflects a greater emphasis on anxieties affiliated to religious or cultural heritage, whereas NAP signifies personal concerns and worries. Further research should investigate this potential distinction because dissimilarities between TPB and NAP are qualitative rather than quantitative. Hence, belief type reflects prevalent mentation rather than exclusive thought. Accordingly, TPB like NAP may have psychopathological elements. Indeed, despite Houran et al. [23] reporting a stronger relationship between schizotypy and NAP, subsequent studies have reported that TPB correlates similarly with schizotypy. The relationship being strongest with the cognitive-perceptual factor that includes productive elements such as magical thinking, odd beliefs, ideas of reference, unusual perceptual experiences, and paranoid ideation [50, 71, 77].

Nonetheless, the two-factor RPBS model provides useful clinical insights for practitioners working with clients who report religious, spiritual, and supernatural problems. Explicitly, TPB and NAP suggest the possible origin of issues and the starting point of therapy/treatment. Specifically, they advise that individuals with elevated levels of TPB and stress would benefit from exposure to approach coping strategies. These together with techniques that promote an internal locus of control may enhance coping and reduce distress. Certainly, follow-up work should investigate relationships between these factors. This is vital because although paranormal belief may not itself be predictive of lower well-being, it may indirectly reflect reduced psychological functioning. From this perspective, like conspiracy theory endorsement, in extreme instances heightened endorsement of paranormal belief could be symptomatic of non-adaptive coping [70].

## Supporting information

S1 Table Traditional paranormal belief and new age philosophy items from the revised paranormal belief scale. (DOCX)

## References

[1] Dagnall NA, Drinkwater K, Parker A, Clough P. Paranormal experience, belief in the paranormal and anomalous beliefs. Paranthropology: Journal of Anthropological Approaches to the Paranormal. 2016 Jul 13;7(1):4–15.

[2] Silva T, Woody A. Supernatural sociology: Americans’ beliefs by race/ethnicity, gender, and education. Socius. 2022 Mar 8; 23780231221084775. https://doi.org/10.1177%2F23780231221084775

[3] Corcoran KE, Scheitle CP, DiGregorio BD. Paranormal Beliefs, Vaccine Confidence, and COVID-19 Vaccine Uptake. Sociology of Religion. 2023 Jun 1;84(2):111–143. 10.1093/socrel/srac024

[4] Rizeq J, Flora DB, Toplak ME. An examination of the underlying dimensional structure of three domains of contaminated mindware: Paranormal beliefs, conspiracy beliefs, and anti-science attitudes. Thinking & Reasoning. 2021 Apr 3;27(2):187–211. 10.1002/acp.3042

[5] Lobato E, Mendoza J, Sims V, Chin M. Examining the relationship between conspiracy theories, paranormal beliefs, and pseudoscience acceptance among a university population. Applied Cognitive Psychology. 2014 Sep;28(5):617–625. 10.1089/acm.2014.0229

[6] Clobert M, Saroglou V, Van Pachterbeke M. Who turns to acupuncture? The role of mistrust of rationality and individualist success. The Journal of Alternative and Complementary Medicine. 2015 Aug 1;21(8):466–471. doi: 10.1089/acm.2014.0229 26090703

[7] Drinkwater K, Dagnall N, Parker A. Reality testing, conspiracy theories, and paranormal beliefs. Journal of Parapsychology. 2012 Mar 1;76(1):57–77.

[8] Irwin HJ. The psychology of paranormal belief: A researcher’s handbook. University of Hertfordshire Press; 2009.

[9] Drinkwater KG, Dagnall N, Denovan A, Williams C. Paranormal belief, thinking style and delusion formation: a latent profile analysis of within-individual variations in experience-based paranormal facets. Frontiers in Psychology. 2021 Jun 28;12:670959. doi: 10.3389/fpsyg.2021.670959 34262510 PMC8273333

[10] Irwin HJ, Dagnall N, Drinkwater K. Paranormal belief and biases in reasoning underlying the formation of delusions. Australian Journal of Parapsychology. 2012 Jun;12(1):7–21.

[11] Irwin HJ, Dagnall N, Drinkwater K. Paranormal beliefs and cognitive processes underlying the formation of delusions. Australian Journal of Parapsychology. 2012 Dec;12(2):107–126.

[12] Dagnall N, Parker A, Munley G, Drinkwater K. Common paranormal belief dimensions. Journal of Scientific Exploration. 2010 Sep 1;24(3):431–477.

[13] Thalbourne MA, Delin PS. A new instrument for measuring the sheep-goat variable: its psychometric properties and factor structure. Journal of the Society for Psychical Research. 1993;59(832):172–186.

[14] Tobacyk JJ. A Revised Paranormal Belief Scale. International Journal of Transpersonal Studies. 2004 Jan 1;23(1):94–98. 10.24972/ijts.2004.23.1.94

[15] Drinkwater K, Denovan A, Dagnall N, Parker A. An assessment of the dimensionality and factorial structure of the Revised Paranormal Belief Scale. Frontiers in Psychology. 2017 Sep 26;8:1693. doi: 10.3389/fpsyg.2017.01693 29018398 PMC5622942

[16] Drinkwater K, Denovan A, Dagnall N, Parker A. The Australian Sheep-Goat Scale: An evaluation of factor structure and convergent validity. Frontiers in Psychology. 2018 Aug 28;9:1594. doi: 10.3389/fpsyg.2018.01594 30210415 PMC6121071

[17] Lange R, Irwin HJ, Houran J. Top-down purification of Tobacyk’s Revised Paranormal Belief Scale. Personality and Individual Differences. 2000 Jul 1;29(1):131–156. 10.1016/S0191-8869(99)00183-X

[18] Rasch G. Probabilistic model for some intelligence and achievement tests. Denmark: Danish Institute for Educational Research; 1960.

[19] Ember CR, Ember M. Anthropology (5th ed.). Englewood Cliffs, NJ: Prentice Hall. (1988).

[20] Peoples J, Bailey G. Humanity: An introduction to cultural anthropology. Cengage Learning; 2014.

[21] Goode E. Paranormal beliefs: A sociological introduction. Waveland Press; 2000.

[22] Irwin HJ. Origins and functions of paranormal belief: the role of childhood trauma and interpersonal control. Journal of the American Society for Psychical Research. 1992;86(3):199–208

[23] Houran J, Irwin HJ, Lange R. Clinical relevance of the two-factor Rasch version of the Revised Paranormal Belief Scale. Personality and Individual Differences. 2001 Aug 1;31(3):371–382. 10.1016/S0191-8869(00)00143-4

[24] Raine A, Reynolds C, Lencz T, Scerbo A, Triphon N, Kim D. Cognitive-perceptual, interpersonal, and disorganized features of schizotypal personality. Schizophrenia Bulletin. 1994 Jan 1;20(1):191–201. doi: 10.1093/schbul/20.1.191 8197415

[25] Dagnall N, Parker A, Munley G. Superstitious belief-negative and positive superstitions and psychological functioning. European Journal of Parapsychology. 2007;22(2):121–137.

[26] Dagnall N, Parker A, Munley G. Assessing superstitious belief. Psychological Reports. 2009 Apr;104(2):447–454. doi: 10.2466/PR0.104.2.447-454 19610474

[27] Dagnall N, Denovan A, Drinkwater KG. Variations in well-being as a function of paranormal belief and psychopathological symptoms: A latent profile analysis. Frontiers in Psychology. 2022 Jun 24;13:886369. doi: 10.3389/fpsyg.2022.886369 35814073 PMC9263512

[28] Dagnall N, Denovan A, Drinkwater KG, Escolà-Gascón Á. Paranormal belief and well-being: The moderating roles of transliminality and psychopathology-related facets. Frontiers in Psychology. 2022 Aug 15;13:915860. doi: 10.3389/fpsyg.2022.915860 36046418 PMC9421129

[29] Dagnall N, Denovan A, Drinkwater KG. Paranormal belief, cognitive-perceptual factors, and well-being: A network analysis. Frontiers in Psychology. 2022 Sep 15;13:967823. doi: 10.3389/fpsyg.2022.967823 36186327 PMC9521162

[30] Thalbourne MA. Transliminality: Further correlates and a short measure. Journal of the American Society for Psychical Research. 1998 Oct;92(4): 402–419.

[31] Carson SH. Creativity and psychopathology: A shared vulnerability model. The Canadian Journal of Psychiatry. 2011 Mar;56(3):144–153. doi: 10.1177/070674371105600304 21443821

[32] Peters E, Day S, McKenna J, Orbach G. Delusional ideation in religious and psychotic populations. British Journal of Clinical Psychology. 1999 Mar;38(1):83–96. doi: 10.1348/014466599162683 10212739

[33] Golden-Kreutz DM, Browne MW, Frierson GM, Andersen BL. Assessing stress in cancer patients: A second-order factor analysis model for the Perceived Stress Scale. Assessment. 2004 Sep;11(3):216–223. doi: 10.1177/1073191104267398 15358877 PMC2746492

[34] Cohen S, Williamson G. Perceived stress in a probability sample of the U.S. In: Spacapam S, Oskamp S, editors. The social psychology of health: Claremont symposium on applied social psychology, Newbury Park: Sage; 1988. pp. 31–67.

[35] Denovan A, Dagnall N, Dhingra K, Grogan S. Evaluating the Perceived Stress Scale among UK university students: Implications for stress measurement and management. Studies in Higher Education. 2019 Jan 2;44(1):120–133. 10.1080/03075079.2017.1340445

[36] Keinan G. The effects of stress and desire for control on superstitious behavior. Personality and Social Psychology Bulletin. 2002 Jan;28(1):102–108. 10.1177/014616720228100

[37] Lasikiewicz N. Perceived stress, thinking style, and paranormal belief. Imagination, Cognition and Personality. 2016 Mar;35(3):306–320. doi: 10.1177/0276236615595235

[38] Keinan G. Effects of stress and tolerance of ambiguity on magical thinking. Journal of Personality and Social Psychology. 1994 Jul;67(1):48–55. doi: 10.1037/0022-3514.67.1.48

[39] Irwin HJ. Belief in the paranormal: A review of the empirical literature. Journal of the American Society for Psychical Research. 1993 Jan 1;87(1):1–39.

[40] Irwin HJ. Paranormal beliefs and the maintenance of assumptive world views. Journal of the Society for Psychical Research. 2003 67:18–25.

[41] Padgett VR, Jorgenson DO. Superstition and economic threat: Germany, 1918–1940. Personality and Social Psychology Bulletin. 1982 Dec;8(4):736–741. 10.1177/0146167282084021

[42] Roe CA, Bell C. Paranormal belief and perceived control over life events. Journal of the Society for Psychical Research. 2016 Apr 26;80(2):65–77.

[43] Cohen S, Kamarck T, Mermelstein R. A global measure of perceived stress. Journal of Health and Social Behavior. 1983 Dec 1:385–396. 10.2307/2136404 6668417

[44] Hewitt PL, Flett GL, Mosher SW. The Perceived Stress Scale: Factor structure and relation to depression symptoms in a psychiatric sample. Journal of Psychopathology and Behavioral Assessment. 1992 Sep;14:247–257. 10.1007/BF00962631

[45] Mimura C, Griffiths P. A Japanese version of the perceived stress scale: translation and preliminary test. International Journal of Nursing Studies. 2004 May 1;41(4):379–385. doi: 10.1016/j.ijnurstu.2003.10.009 15050849

[46] Medvedev ON, Krägeloh CU, Hill EM, Billington R, Siegert RJ, Webster CS, et al. Rasch analysis of the Perceived Stress Scale: Transformation from an ordinal to a linear measure. Journal of Health Psychology. 2019 Jul;24(8):1070–1081. doi: 10.1177/1359105316689603 28810395

[47] Salak B, Lindberg K, Kienast F, Hunziker M. Hybrid choice model dataset of a representative Swiss online panel survey on peoples’ preferences related to mixed renewable energy scenarios in landscapes and the effect of landscape-technology fit. Data in Brief. 2021 Jun 1;36:107025. doi: 10.1016/j.dib.2021.107025 34026963 PMC8131564

[48] Kees J, Berry C, Burton S, Sheehan K. An analysis of data quality: Professional panels, student subject pools, and Amazon’s Mechanical Turk. Journal of Advertising. 2017 Jan 2;46(1):141–155. 10.1080/00913367.2016.1269304

[49] Dagnall N, Drinkwater K, Denovan A, Parker A, Rowley K. Misperception of chance, conjunction, framing effects and belief in the paranormal: A further evaluation. Applied Cognitive Psychology. 2016 May;30(3):409–419. 10.1002/acp.3217

[50] Denovan A, Dagnall N, Drinkwater K, Parker A. Latent profile analysis of schizotypy and paranormal belief: Associations with probabilistic reasoning performance. Frontiers in Psychology. 2018 Jan 26;9:35. doi: 10.3389/fpsyg.2018.00035 29434562 PMC5791384

[51] Irwin HJ, Dagnall N, Drinkwater K. The role of doublethink and other coping processes in paranormal and related beliefs. Journal of the Society for Psychical Research. 2015 Apr 1;79(2):80–97.

[52] Krishnaveni R, Deepa R. Controlling common method variance while measuring the impact of emotional intelligence on well-being. Vikalpa. 2013 Jan;38(1):41–48. 10.1177/0256090920130104

[53] Kline RB. Principles and practice of structural equation modeling. Guilford publications; 2023 May 24.

[54] Hu LT, Bentler PM. Cutoff criteria for fit indexes in covariance structure analysis: Conventional criteria versus new alternatives. Structural equation modeling: A multidisciplinary Journal. 1999 Jan 1;6(1):1–55. 10.1080/10705519909540118

[55] Browne MW. Alternative ways of assessing model fit. Testing structural equation models. 1993.

[56] Akaike H. A new look at the statistical model identification. IEEE transactions on automatic control. 1974 Dec;19(6):716–723. 10.1109/TAC.1974.1100705

[57] Byrne BM. Structural equation modeling with Mplus: Basic concepts, applications, and programming. Routledge; 2013 Jun 17.

[58] Gignac GE, Szodorai ET. Effect size guidelines for individual differences researchers. Personality and Individual Differences. 2016 Nov 1;102:74–78. 10.1016/j.paid.2016.06.069

[59] Dagnall N, Denovan A, Drinkwater K, Parker A, Clough P. Statistical bias and endorsement of conspiracy theories. Applied Cognitive Psychology. 2017 Jul;31(4):368–378. 10.1002/acp.3331

[60] Hair JF, Anderson RE, Tatham RL, Black WC. Multivariate Data Analysis New Jersey.

[61] Raykov T. Analytic estimation of standard error and confidence interval for scale reliability. Multivariate Behavioral Research. 2002 Jan 1;37(1):89–103. doi: 10.1207/S15327906MBR3701_04 26824170

[62] Diamantopoulos A, Siguaw JA. Introducing LISREL: A guide for the uninitiated. Sage; 2000 Sep 22.

[63] Drinkwater KG, Denovan A, Dagnall N. Paranormal belief, psychopathological symptoms, and well-being: Latent profile analysis and longitudinal assessment of relationships. Plos one. 2024 Mar 6;19(3):e0297403. doi: 10.1371/journal.pone.0297403 38446771 PMC10917279

[64] Irwin HJ. Belief in the paranormal and a sense of control over life. European Journal of Parapsychology. 2000;15:68–78.

[65] Mowen TJ, Heitkamp A, Boman J. Paranormal activity: Self-control theory and belief in the paranormal. Deviant Behavior. 2022 Jun 3;43(6):728–742. 10.1080/01639625.2021.1915723

[66] Lindeman M, Aarnio K. Paranormal beliefs: Their dimensionality and correlates. European Journal of Personality. 2006 Nov;20(7):585–602. 10.1002/per.60

[67] Lindeman M, Aarnio K. Superstitious, magical, and paranormal beliefs: An integrative model. Journal of Research in Personality. 2007 Aug 1;41(4):731–744. 10.1016/j.jrp.2006.06.009

[68] Mackay J, Charles ST, Kemp B, Heckhausen J. Goal striving and maladaptive coping in adults living with spinal cord injury: Associations with affective well-being. Journal of Aging and Health. 2011 Feb;23(1):158–176. doi: 10.1177/0898264310382039 20876363

[69] Callaghan AJ, Irwin HJ. Paranormal belief as a psychological coping mechanism. Journal of the Society for Psychical Research. 2003. 67(3), 200–207.

[70] Marchlewska M, Green R, Cichocka A, Molenda Z, Douglas KM. From bad to worse: Avoidance coping with stress increases conspiracy beliefs. British Journal of Social Psychology. 2022 Apr;61(2):532–549. doi: 10.1111/bjso.12494 34462919

[71] Dagnall N, Denovan A, Drinkwater K, Parker A, Clough P. Toward a better understanding of the relationship between belief in the paranormal and statistical bias: The potential role of schizotypy. Frontiers in Psychology. 2016 Jul 14;7:1045. doi: 10.3389/fpsyg.2016.01045 27471481 PMC4943933

[72] Ryff CD. Happiness is everything, or is it? Explorations on the meaning of psychological well-being. Journal of Personality and Social Psychology. 1989 Dec;57(6):1069–1081. 10.1037/0022-3514.57.6.1069

[73] Irwin HJ, Marks AD, Geiser C. Belief in the Paranormal: A State, or a Trait?. The Journal of Parapsychology. 2018 Mar 22;82(1):24–41.

[74] Spector PE. Do not cross me: Optimizing the use of cross-sectional designs. Journal of Business and Psychology. 2019 Apr 15;34(2):125–137. 10.1007/s10869-018-09613-8

[75] Ashton MC, Lee K. The HEXACO model of personality structure and the importance of the H factor. Social and Personality Psychology Compass. 2008 Sep;2(5):1952–1962. 10.1111/j.1751-9004.2008.00134.x

[76] Baker JO, Bader CD, Mencken FC. A bounded affinity theory of religion and the paranormal. Sociology of Religion: A Quarterly Review. 2016 Dec 1;77(4):334–358. 10.1093/socrel/srw040

[77] Dagnall N, Denovan A, Drinkwater K, Parker A, Clough PJ. Urban legends and paranormal beliefs: the role of reality testing and schizotypy. Frontiers in psychology. 2017 Jun 8;8:262250. doi: 10.3389/fpsyg.2017.00942 28642726 PMC5463090

