# Supplementary material for: Re-evaluation of the relationship between paranormal belief and perceived stress using statistical modelling
Source: PLoS One. 2024 Nov 13;19(11):e0312511. doi: 10.1371/journal.pone.0312511 (PMC11559985; doi:10.1371/journal.pone.0312511)
Supplement: S1 Table — (DOCX) [file pone.0312511.s001.docx]

S1 Table. Traditional Paranormal Belief and New Age Philosophy items from the Revised Paranormal Belief Scale.

| Scale | Item |
| --- | --- |
| Traditional Paranormal Belief | 8 - There is a devil. |
|  | 17 - Through the use of formulas and incantations, it is possible to cast spells on persons. |
|  | 22 - There is a heaven and a hell. |
|  | 24 - There are actual cases of witchcraft. |
|  | 26 - Some people have an unexplained ability to predict the future. |
| New Age Philosophy | 2 - Some individuals are able to levitate (lift) objects through mental forces. |
|  | 3 - Black magic really exists. |
|  | 5 - Your mind or soul can leave your body and travel (astral projection). |
|  | 7 - Astrology is a way to accurately predict the future. |
|  | 9 - Psychokinesis, the movement of objects through psychic powers, does exist. |
|  | 12 - During altered states, such as sleep or trances, the spirit can leave the body |
|  | 14 - The horoscope accurately tells a person’s future. |
|  | 16 - A person’s thoughts can influence the movement of a physical object. |
|  | 19 - Reincarnation does occur. |
|  | 21 - Some psychics can accurately predict the future. |
|  | 23 - Mind reading is not possible. |
